# Propofol Inhibits the Activation of p38 through Up-Regulating the Expression of Annexin A1 to Exert Its Anti-Inflammation Effect

**DOI:** 10.1371/journal.pone.0027890

**Published:** 2011-12-02

**Authors:** Jing Tang, Xi Chen, Weifeng Tu, Yuanbo Guo, Zhenlong Zhao, Qiong Xue, Chunshui Lin, Jinfang Xiao, Xuegang Sun, Tao Tao, Miaoning Gu, Youtan Liu

**Affiliations:** 1 Department of Anesthesia, Nanfang Hospital, Southern Medical University, Guangzhou, People's Republic of China; 2 Department of Anesthesia, Guangzhou General Hospital of Guangzhou Military Command, Guangzhou, People's Republic of China; 3 The Key Laboratory of Molecular Biology, State Administration of Traditional Chinese Medicine, School of Traditional Chinese Medicine, Southern Medical University, Guangzhou, People's Republic of China; 4 Department of Anesthesia, Shenzhen Hospital, The University of Hong Kong, Shenzhen, People's Republic of China; University of Nebraska Medical Center, United States of America

## Abstract

Inflammatory response is a kind of nonspecific immune response, with the central link of vascular response, which is mainly manifested by changes in neutrophils and vascular endothelial cells. In recent years, the in vivo and in vitro role of intravenous anesthetic propofol in inhibiting inflammatory response has been attracting more and more attention, but the anti-inflammatory mechanisms of propofol for mononuclear cells still remain undefined. In this study, proteomics analysis was applied to investigate protein expression profile changes in serum mononuclear cells following intervention of rats with endotoxemia using propofol. After two-dimensional electrophoresis and mass spectrometric identification, it has been found that the protein Annexin A1 was up-regulated in the propofol intervention group. Annexin A1 is a glucocorticoid-dependent anti-inflammatory protein. After detection using ELISA and Western blot assays, it has also been found that propofol can not only promote the expression of Annexin A1, but also inhibit the phosphorylation level of p38 and release of inflammatory factors (IL-1β, IL-6 and TNF-α) in rats with endotoxemia. In order to further determine the role of up-regulated expression of Annexin A1 in anti-inflammation of propofol, this gene was silenced in vitro in human THP-1 cells, to detect the phosphorylation status of p38 and release of inflammatory factors. The results show that Annexin A1 can negatively regulate phosphorylation of p38 and release of IL-1β, IL-6 and TNF-α in THP-1 cells following propofol intervention and lipopolysaccharide (LPS) stimulation. Our results clearly indicate that propofol can up-regulate Annexin A1 to inhibit the phosphorylation level of p38 and release of IL-1β, IL-6 and TNF-α, so as to inhibit inflammatory response. Therefore, it can be speculated that Annexin A1 might be the key signaling protein in the in vivo and in vitro anti-inflammatory mechanisms of propofol.

## Introduction

Sepsis is a kind of systemic inflammatory response syndrome caused by a variety of pathogenic microorganisms or their toxins [1], [2]. Severe sepsis can lead to multiple organ dysfunction syndrome, which accounts for one of the major causes of high mortality in ICU patients [3], [4]. Endotoxin is one of the structural constitutions of the outer membrane of the cell wall in Gram-negative bacteria. Its chemical essence is LPS, which is the major factor inducing sepsis [5]. LPS can extensively act on a variety of tissues and organs in the body [5]. Mononuclear cells, neutrophils and endothelial cells are the major effector cells [6], [7]. Therefore, studies on the molecular mechanisms of sepsis or endotoxemia mainly concentrate on the three effector cells.

Propofol is an intravenous general anesthetic [8]. Due to its fast onset of action, in addition to its characteristics of regaining full consciousness rapidly without accumulation during continuous infusion, it is widely used in anesthesia induction and maintenance, as well as sedation for ICU patients [9]. In recent years, the anti-inflammatory role of propofol has gradually attracted people's interest [10]–[14]. Many studies have shown that propofol can: ① inhibit neutrophil adhesion to vascular endothelial cells [15]; ② scavenge oxygen free radicals and inhibit oxidative damage [16]; ③ inhibit the release of inflammatory factors like TNF-α, IL-6 and IL-β [16]–[18]. Though there has been a common understanding on the role of propofol in inhibiting inflammatory response, the specific mechanism still remains unclear and there have been no complete theoretical basis to guide clinical practice, which greatly limits the application of propofol in the anti-inflammatory domain.

In this study, the impact of propofol on microcirculation of the model of LPS-stimulated inflammation was investigated using proteomics analysis from effector cells-mononuclear cells of sepsis, including the impact on the serum cytokine profile and the protein expression profile in serum mononuclear cells, to explore the key signaling molecules mediating the anti-inflammatory effect of propofol and the signaling pathway. Mononuclear cells were further verified for in-depth study of the anti-inflammatory mechanism of propofol for mononuclear cells.

## Results

### Quantitative comparison and identification of protein spots on 2D gels

**Figure 1:**
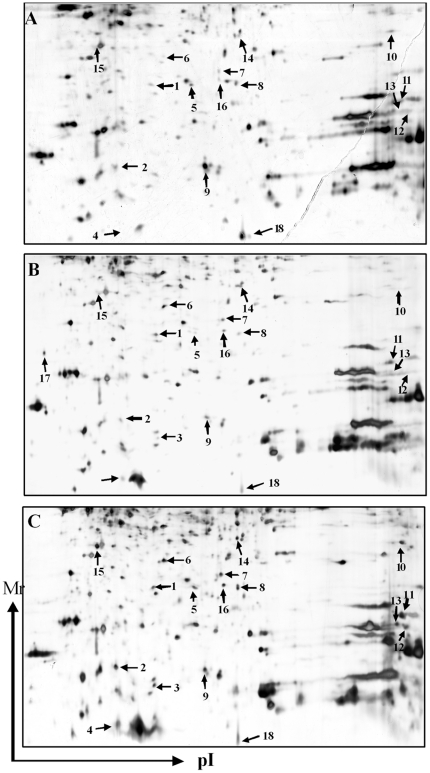
Representative 2D gels. Control group(upper), LPS+Intralipid group(middle) and LPS+propofol group(lower). Fifteen of these protein spots were successfully identified by MALDI-TOF MSMS and by subsequent comparative sequence search in the Mascot database.

To determine the change of monocyte proteins profile in response to LPS and propofol, gel-based comparative proteomic analysis was performed. As shown in Fig. 1, eighteen protein spots were found to be significantly altered. Fifteen of these protein spots were successfully identified by MALDI-TOF MSMS and by subsequent comparative sequence search in the Mascot database (Table 1). Among them, destrin and glutathione peroxidase 1 were substantially up-regulated in the control, LPS and LPS+propofol groups. UMP-CMP kinase and myosin-9 had the lowest expression in control group; While cofilin-1, ATP synthase subunit alpha, mitochondrial precursor and hemoglobin subunit alpha had the lowest expression in LPS group. In addition, the expression of protein S100-A8, L-lactate dehydrogenase A chain, peptidyl-prolyl cis-trans isomerase B precursor, peptidyl-prolyl cis-trans isomerase, mitochondrial precursor and Annexin A1 increased dramatically only in LPS+propofol group. Incontrast, the expression of ubiquitin reached to highest level just in control group, and S-phage kinase-associated protein 1 emerged only in LPS group.

**Table 1:** List of Identified Protein Spots Differentially Expressed in control, LPS, and LPS plus propofol groups.

spot	theoretical*Mr* (kDa)	totalscore	queriesmached	SwissProtID	Protein description
1	22.44	152	100	Q4KM73	UMP-CMP kinase
2	18.75	121	100	P45592	Cofilin-1
3	19.81	30	94.68	QAT7M0E3	Destrin
4	10.29	126	100	PA5T0115	Protein S100-A8
5	59.83	306	100	PT15999	ATP synthase subunit alpha, mitochondrial precursor
6	227.57	194	100	QAT62812	Myosin-9
8	15.23	69	99.99	PG1A9014	Hemoglobin subunit alpha
10	36.71	205	100	PT04642	L-lactate dehydrogenase A chain
11	22.85	64	99.95	P24368	Peptidyl-prolyl cis-trans isomerase B precursor
13	22.14	248	100	P29117	Peptidyl-prolyl cis-trans isomerase, mitochondrial precursoe
14	16.08	354	100	PT02091	Hemoglobin subunit beta-1
15	39.15	691	100	P07150	Annexin A1
16	22.46	230	99.99	PT04041	Glutathione peroxidase 1
17	18.83	228	100	QAT6PEC4	S-phage kinase-associated protein 1
18	8.56	320	100	PT62989	Ubiquitin

### Verification of up-regulation of Annexin A1 in LPS+propofol group by Western blot in endotoxaemic rats

**Figure 2:**
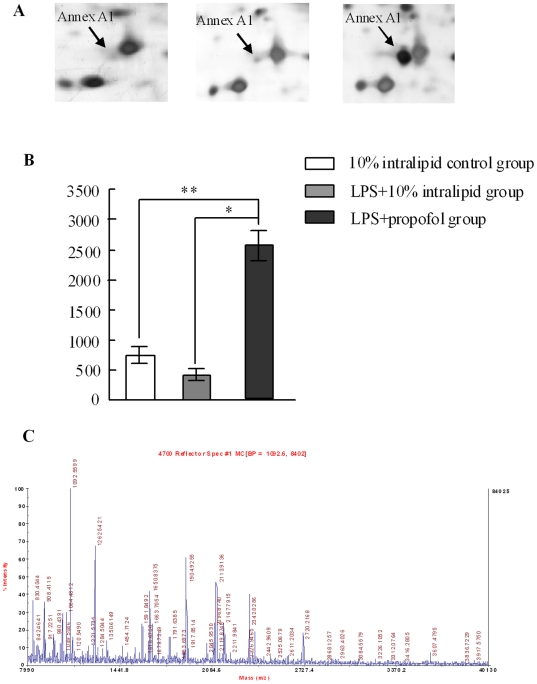
Identification of Annexin A1 up-regulation in LPS+propofol group. A. Gels in control(1), LPS(2), and LPS+propofol(3) group has been enlarged to show the high expression of Annex A1 in three groups. B. The densitometric analysis of each protein was calculated from 9 different gels using PDQuest software. Each bar represents the mean ± SD of intensity, the expression of Annex A1 in LPS+prpofol group increased dramatically compared with LPS+Intralipid group(*p<0.001) and control Group(**p<0.001). C. MSMS of in-gel trypsin digests of the protein and analysis of the depicted peptide spectrum resulted in the identification of Annex A1.

**Figure 3:**
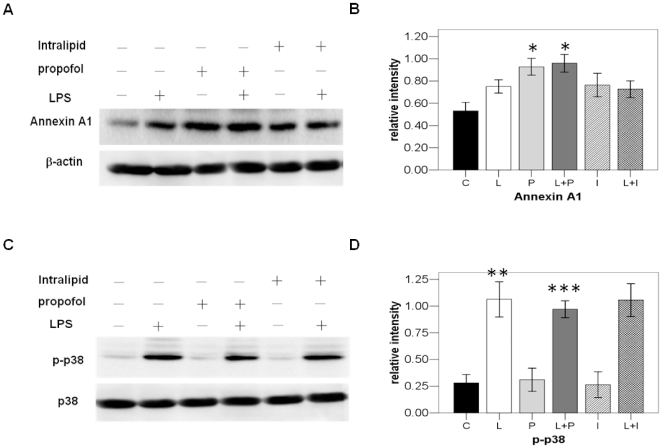
Confirming the expression of Annexin A1 and phos-p38 in monocytes with Western Blot assay. A. Monocytes lysis samples from the individual rats were separated on 12% SDS-PAGE gels and transferred to PVDF membranes for Western blotting analysis. B. Gray intensity analysis of the western blot results of six groups. Each bar represents the mean ± S.D, (*p<0.01, n = 6, compared with the other four groups) C group: control group; L group: LPS group; P group: propofol group; L+P group: LPS+propofol group; I group: intralipid group; L+I group: LPS+intralipid group. C. Monocytes lysis samples from the individual rats were separated on 12% SDS-PAGE gels and transferred to PVDF membranes for Western blotting analysis. D. Gray intensity analysis of the western blot results of four groups. Each bar represents the mean ± S.D, (**p<0.01, n = 6, compared with the C, P and I groups; ***p<0.05, n = 6, compared with the L and L+I groups) C group: control group; L group: LPS group; P group: propofol group; L+P group: LPS+propofol group; I group: intralipid group; L+I group: LPS+intralipid group.

As gel-based proteomic analysis showed that the expression of Annexin A1 increased dramatically in LPS+propofol group, Annexin A1 was elected for further investigation. The corresponding differential expression patterns identified by 2D electrophoresis and the MALDI-TOF mass spectra are shown in Fig. 2. Annexin A1 is a 37 000 molecular weight anti-inflammatory protein induced by glucocorticoids and mediates some of the beneficial actions of glucocorticoids, such as inhibition of cellular proliferation [19], anti-inflammatory effects [20]–[22], the regulation of cell differentiation [23] and membrane trafficking [24], [25]. To more rigorously study the effect of propofol on the expression of Annexin A1 in monocytes, 36 animals were divided into six groups control group, LPS group, propofol group, LPS+propofol group, intralipid group and LPS+intralipid. Annexin A1 expression in monocytes of each group was determined by Western blot. As shown in Fig. 3A, the amount of Annexin A1 in monocytes showed no statistical difference both in control, intralipid, and LPS groups(P>0.05). However, in the monocytes of propofol and LPS+propofol group, the expression of Annexin A1 increased dramatically in accordance with our 2D gels results. These results indicated that propofol can promote the expression of Annexin A1 in the monocytes of endotoxaemic rats. The total β-actin loaded on SDS-PAGE was used as internal control (Fig. 3B).

### Propofol inhibits the activation of p38 and release of IL-1β, IL-6, and TNF-α in endotoxaemic rats

**Figure 4:**
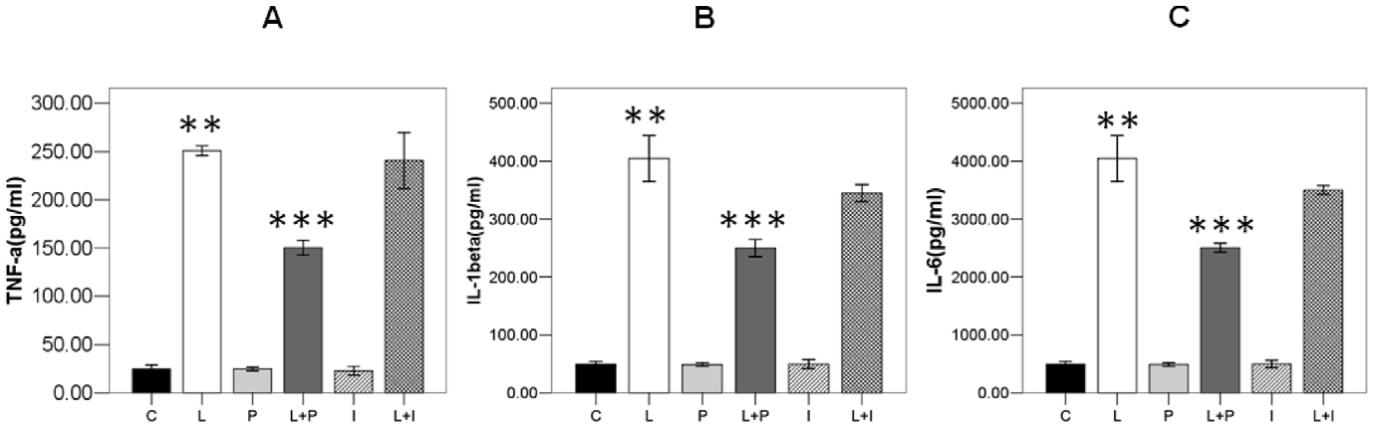
IL-1β, IL-6 and TNF-α quantitization in the serum of six groups. A. ELISA confirmation of TNF-α in the serum of six groups. Each bar represents the mean ± S.D, significant differences were found between L group and L+P Group(*p<0.01, n = 6). B. ELISA confirmation of IL-1β in the serum of four groups. Each bar represents the mean ± S.D, significant differences were found between L group and L+P Group(*p<0.01, n = 6). C. ELISA confirmation of IL-6 in the serum of four groups. Each bar represents the mean ± S.D, significant differences were found between L group and L+P Group (*p<0.01, n = 6).

As it has been reported that Annexin A1 is a negative regulator of IL-1β, IL-6, and TNF-α and has inhibitory effects on the activation of p38 MAPK signal transduction pathway(12). Our 2D and Western Blot results showed that in endotoxaemic rats, propofol promotes the expression of Annexin A1 of monocytes in the blood. Therefore, we further studied the effect of propofol on the activation of p38 and release of IL-1β, IL-6, and TNF-α in endotoxaemic rats. Without the treatment of LPS, the phosphorylation of p38 remains in a low basal levels both in intralipid and propofol group. LPS dramatically increased the phosphorylation level of p38 in the monocytes, and this effect could be partially inhibited by propofol(Fig. 3C). The total p38 and β-actin loaded on SDS-PAGE was used as internal control (Fig. 3B). Then the sera amount of IL-1β, IL-6, and TNF-α were measured by ELISA in all the four groups. As shown in Fig. 4, sera level of IL-1β, IL-6, and TNF-α showed no difference between intralipid control and propofol group. LPS could obviously increase the amount of IL-1β, IL-6, and TNF-α in the serum. This effect of LPS could also be partially inhibited by propofol.

### The impact of propofol on Annexin A1 and phosphorylation of p38 following LPS stimulation was validated and the time effects

**Figure 5:**
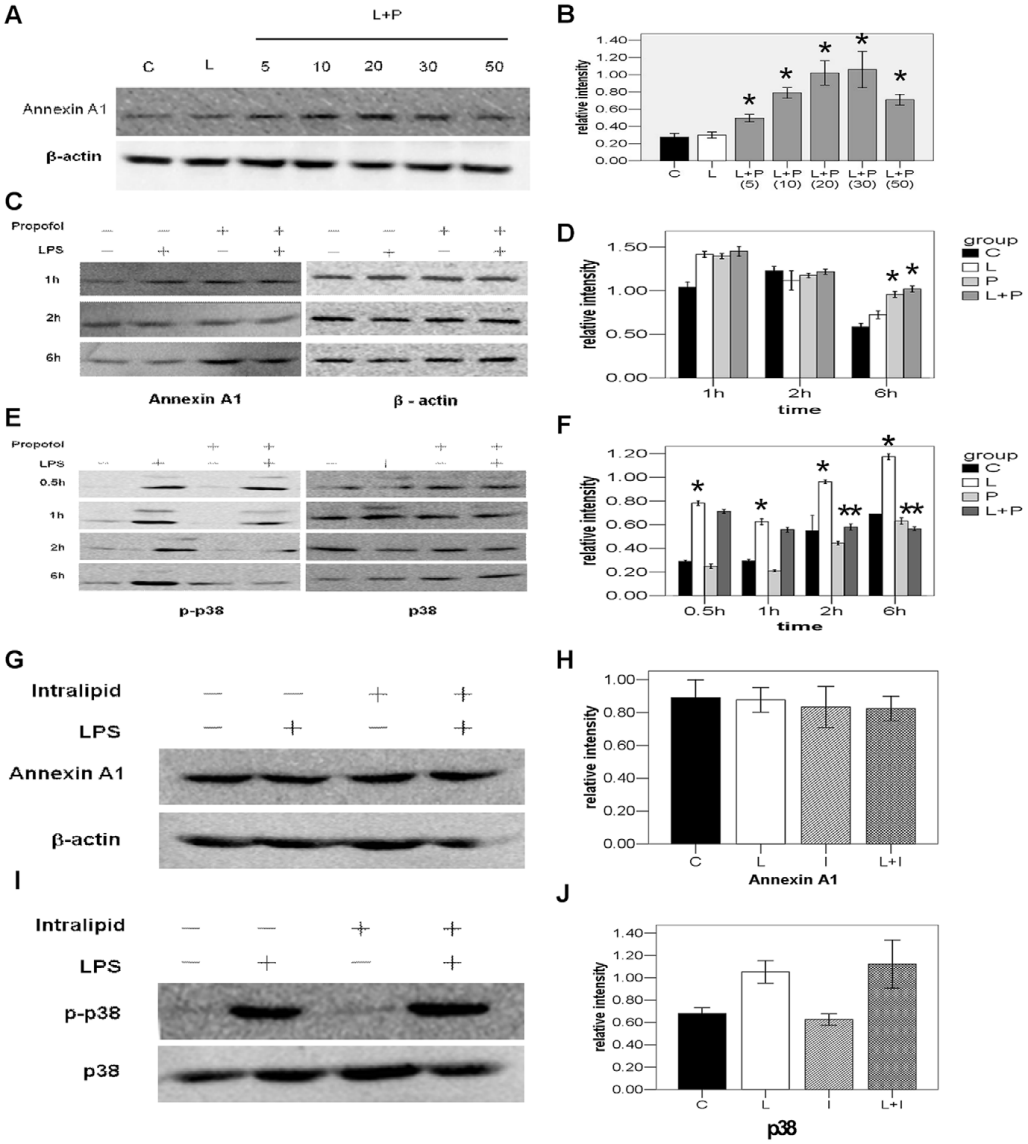
The impact of propofol on Annexin A1 and phosphorylation of p38 following LPS stimulation was validated and the time and concentration effects were analyzed with Western Blot. A. Lysis samples of THP-1 cells from 5 µg/ml to 50 µg/ml of propofol treated were separated on 12% SDS-PAGE gels and transferred to PVDF membranes for Western blotting analysis of Annexin A1. B. Gray intensity analysis of the western blot results of seven groups. Each bar represents the mean ± S.D, (*p<0.01, n = 3, compared with C and L groups in 6 h) C group: control group; L group: LPS group; L+P(5, 10, 20, 30, 50) group: LPS+different concentration (5, 10, 20, 30, 50 µg/ml)propofol group. C. Lysis samples of THP-1 cells from 1 h to 6 h were separated on 12% SDS-PAGE gels and transferred to PVDF membranes for Western blotting analysis of Annexin A1. D. Gray intensity analysis of the western blot results of four groups. Each bar represents the mean ± S.D, (*p<0.01, n = 3, compared with C and L groups in 6 h) C group: control group; L group: LPS group; P group: propofol group; L+P group: LPS+propofol group. E. Lysis samples of THP-1 cells from 1 h to 6 h were separated on 12% SDS-PAGE gels and transferred to PVDF membranes for Western blotting analysis of phos-p38. F. Gray intensity analysis of the western blot results of four groups. Each bar represents the mean ± S.D, (**p<0.001, compared with the C and P groups in 0.5 h, 1 h, 2 h, 6 h; **p<0.05, compared with the L groups in 2 h and 6 h, n = 3) C group: control group; L group: LPS group; P group: propofol group; L+P group: LPS+propofol group. G. Lysis samples of THP-1 cells were separated on 12% SDS-PAGE gels and transferred to PVDF membranes for Western blotting analysis of Annexin A1. H. Gray intensity analysis of the western blot results of six groups. Each bar represents the mean ± S.D. No significant differences were found between L group and L+I Group (p>0.05). C group: control group; L group: LPS group; I group: intralipid group; L+I group: LPS+intralipid group. I. Lysis samples of THP-1 cells were separated on 12% SDS-PAGE gels and transferred to PVDF membranes for Western blotting analysis of phos-p38. J. Gray intensity analysis of the western blot results of four groups. Each bar represents the mean ± S.D. No significant differences were found between L group and L+I Group (p>0.05). C group: control group; L group: LPS group; I group: intralipid group; L+I group: LPS+intralipid group.

Human monocytic cell line-THP-1 was selected for in vitro study of the anti-inflammatory mechanism of propofol. First, the concentration impact(treated for 5, 10, 20, 30, 50 µg/ml) of propofol on Annexin A1 was validated. Propofol treatment increased the expression of Annexin A1 in a concentration-dependent manner (Fig. 5A). Since we observed that propofol (20 µg/ml) caused significant effect of Annexin A1 (P<0.01, Fig. 5B), this treatment condition was used in the following experiments. Then the impact of propofol on Annexin A1 and phosphorylation of p38 following LPS stimulation was validated and the time effects(treated for 0.5, 1, 2, 6 h) were analyzed. The results showed that propofol can activate the expression of Annexin A1 no matter there was LPS stimulation or not and the effect was the most obvious at 6 h (Fig. 5C). Correspondingly, propofol can inhibit LPS-induced increase in the phosphorylation level of p38 and the inhibitory effect on phosphorylation of p38 was significant at 2 h and 6 h following propofol treatment (Fig. 5E). The solvent of propofol is intralipid, which is reported to have the anti-inflammatory effect in many literatures. Thus, after comparison of intralipid and propofol in vitro study of this experiment, it has been found that intralipid can not activate the expression of Annexin A1, nor inhibit the phosphorylation of p38 at 6 h.(Fig. 5J, I)

### The release of IL-1β, IL-6 and TNF-α in the supernatant of LPS-stimulated THP-1 cells in vitro

**Figure 6:**
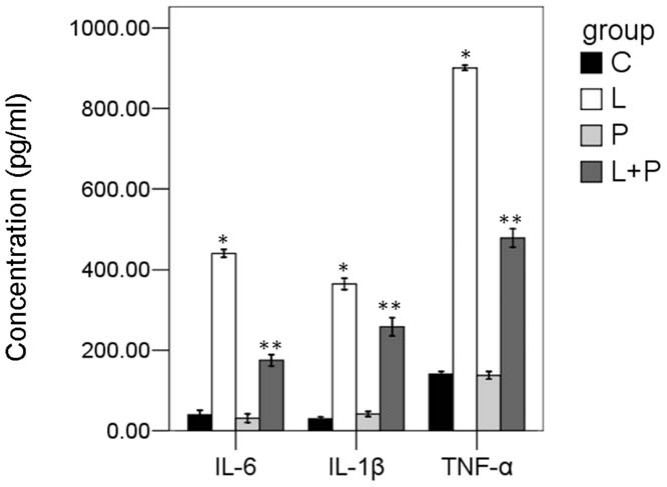
IL-1β, IL-6 and TNF-α quantitization in the serum of THP-1 cells. ELISA confirmation of TNF-α IL-1β, IL-6 in the serum of THP-1 cells. Each bar represents the mean ± S.D, significant differences were found between L group and L+P Group(*p<0.001, compared with C and P groups; **p<0.01, compared with the L groups).

The supernatant of LPS-stimulated THP-1 cells of IL-1β, IL-6, and TNF-α were measured by ELISA in all the four groups. As shown in Fig. 6, supernatant level of IL-1β, IL-6, and TNF-α showed no difference between contrtol control and propofol group (P>0.05). LPS could obviously increase the amount of IL-1β, IL-6, and TNF-α in the supernatant (P<0.01). This effect of LPS could also be partially inhibited by propofol (P<0.05).

### After inhibition of Annexin A1 in THP-1, the expression of phosphorylation of p38 and the release of IL-1β, IL-6 and TNF-α under LPS stimulation and propofol treated

**Figure 7:**
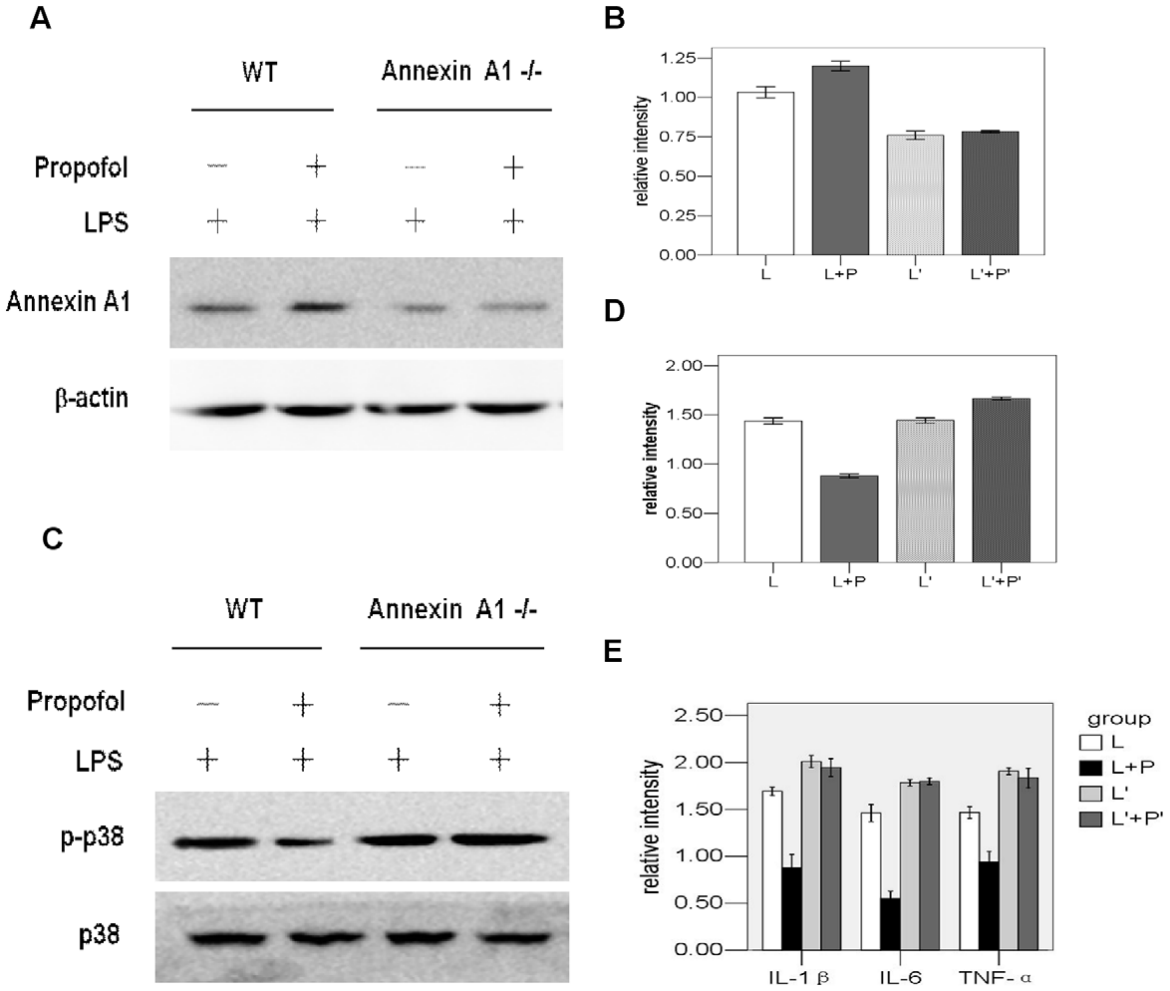
The impact of propofol on phosphorylation of p38 and the release of IL-1β, IL-6 and TNF-α under LPS stimulation after inhibition of Annexin A1 in THP-1. A. Lysis samples of THP-1 cells were separated on 12% SDS-PAGE gels and transferred to PVDF membranes for Western blotting analysis of Annexin A1. B. Gray intensity analysis of the western blot results of six groups. Each bar represents the mean ± S.D. No significant differences were found between L' group and L'+P' Group (p>0.05). L group: LPS group with wide-type Annexin A1; L+P group: LPS+propofol group with wide-type Annexin A1 ; L' group: LPS group with transfected shRNA of Annexin A1; L'+P' group: LPS+propofol group with transfected shRNA of Annexin A1. C. Lysis samples of THP-1 cells were separated on 12% SDS-PAGE gels and transferred to PVDF membranes for Western blotting analysis of phosphorylation of p38. D. Gray intensity analysis of the western blot results of four groups. Each bar represents the mean ± S.D. No significant differences were found between L' group and L'+P' Group (p>0.05). L group: LPS group with wide-type Annexin A1; L+P group: LPS+propofol group with wide-type Annexin A1 ; L' group: LPS group with transfected shRNA of Annexin A1; L'+P' group: LPS+propofol group with transfected shRNA of Annexin A1. E. the experession of mRNA of TNF-α IL-1β, IL-6 in THP-1 cells. Each bar represents the mean ± S.D, no significant differences were found L'+P' group compared with L' group.

To be investigated whether Annexin A1 is the key anti-inflammatory protein of propofol and whether the anti-inflammatory effect of propofol is realized through inhibiting phosphorylation of p38, RNAi technology was applied to detect the impact of propofol on phosphorylation of p38 and the release of IL-1β, IL-6 and TNF-α under LPS stimulation after inhibition of Annexin A1 in THP-1 cells. THP-1 cells were transiently transfected with the shRNA of Annexin A1 by electroporation. Then the lysis of THP-1 cells were separated on 12% SDS-PAGE gels and transferred to PVDF membranes for Western blotting analysis of phosphorylation of p38. Fig. 7C showed that propofol had no impact on phosphorylation of p38 under LPS stimulation following inhibition of Annexin A1 (P>0.05). IL-1β, IL-6, and TNF-α mRNA of LPS-stimulated THP-1 cells were measured by RT-PCR in all the four groups. However, the results showed that the mRNA of IL-1β, IL-6, and TNF-α had no significantly increased treated by LPS+propofol compared to LPS group following inhibition of Annexin A1. (Fig. 7E).

## Discussion

The anti-inflammatory effect of intravenous general anesthetic propofol has gradually attracted people's attention [10]–[14]. Many literatures have reported that propofol can inhibit the release of inflammatory mediators including IL-1β, IL-6 and TNF-α [16], [17], but the specific mechanism of action for propofol to inhibit these inflammatory factors and the signal transduction mechanism are poorly understood, which limits and hinders the specific clinical application of anti-inflammation for propofol. Mononuclear cells are the most important effector cells in systemic inflammatory response [7]. Therefore, a rat model of endotoxemia was established to conduct two-dimensional electrophoresis proteomic analysis of blood mononuclear cells in rats and find inflammation-related proteins regulated by propofol. It was found after mass spectrometry that some inflammation-related proteins such as: S100-A8, Annexin A1 and mitochondrial precursoe and so on were significantly increased in the LPS+propofol treatment group.

Annexin A1 is a member of the membrane calcium protein family with the size of 37 KD [26]. Glucocorticoids can regulate its synthesis and function [27]. Annexin A1 is highly expressed in the cytoplasm of human and rat neutrophils, mononuclear cells and macrophages. Annexin A1 can be rapidly mobilized to the surface of cells for secretion as cells are activated [28]. Some research has shown that Annexin A1 can inhibit inflammatory response [29]–[32]: arthritis has been found in Annexin A1-knockout mice, accompanied by increased expression of IL-1 and IL-6, and leukocyte and IL-1β migration was significantly inhibited also in Annexin A1-knockout mouse models of inflammation, suggesting that Annexin A1 is closely related to inflammatory response.

Thus, Annexin A1 was selected for further validation and further study. Western blot analysis showed from the animal level that in animals with endotoxemia, propofol can increase the expression of Annexin A1 in blood mononuclear cells, which is in line with the result of two-dimensional electrophoresis. Meanwhile, detection of the release of inflammatory factors also showed that propofol can significantly reduce the release of pro-inflammatory factors (TNF-α, IL-1β, IL-6). Therefore, it can be speculated that the anti-inflammatory effect of propofol might be realized through activation of Annexin A1 to reduce the release of inflammatory factors. This is the first time of detecting the correlation between the anti-inflammatory effect of propofol and Annexin A1 at home and abroad.

The mitogen-activated protein kinase (MAPK) is an important intracellular signal transduction system of mediating extracellular signal response in cells by eukaryotic cells [33]. The p38 signal pathway in the MAPK system mainly functions in inflammation. Pro-inflammatory factors, bacterial components and UV irradiation etc can all activate the p38 pathway [34]. The latest research reports that Annexin A1 is an endogenous factor negatively regulating the activity of MAPK and IL-6 [35]. In Annexin A1^−/−^ cells, the phosphorylation level of p38 was increased after IL-1 induction of inflammatory response and after given the p38 inhibitor, the release of IL-6 was reduced. Thus, it should be explored whether the anti-inflammatory effect of propofol can affect phosphorylation of p38. After detecting the phosphorylation level of p38 in blood mononuclear cells in rats with endotoxemia, it has been found that LPS can evidently induce phosphorylation of p38 and propofol treatment can significantly inhibit phosphorylation of p38, prompting that the anti-inflammatory effect of propofol is related with the inhibition of phosphorylation of p38. Nonetheless, it still awaits further investigation whether the inhibitory effect of propofol on phosphorylation of p38 is directly realized through up-regulation of Annexin A1 and whether Annexin A1 is the key protein in the anti-inflammatory mechanisms of propofol.

In the absence of rat monocytic cell lines, human monocytic cell line-THP-1 was selected for in vitro study of the anti-inflammatory mechanism of propofol. First, the impact of propofol on Annexin A1 and phosphorylation of p38 following LPS stimulation was validated and the time effects were analyzed. The results showed that propofol can activate the expression of Annexin A1 no matter there was LPS stimulation or not and the effect was the most obvious at 6 h. Correspondingly, propofol can inhibit LPS-induced increase in the phosphorylation level of p38 and the inhibitory effect on phosphorylation of p38 was significant at 2 h and 6 h following propofol treatment. In the meantime, propofol can reduce the release of inflammatory factors in the supernatant of THP-1 cells under LPS stimulation, which is in line with the result from the animal level and once again confirms that the anti-inflammatory effect of propofol is related to the activation of Annexin A1 and inhibition of p38 phosphorylation.Lecona E et al. [36] showed that Annexin A1 promoter activity and protein expression is increased by activation of p38 MAPK. Thus, Annexin A1 could be regulated by propofol/p38 activation, and Annexin A1 should not be the primary protein during propofol's anti-inflammation. It isn't in line with our research result. However, we find that studied models of this two article are not the same. Lecona E et al. mainly research effects of Annexin A1 and p38 in cell differentiation regulation in human colon adenocarcinoma cells, while this paper highlights effects of Annexin A1 and p38 in inflammatory mechanism of propofol, which is possibly the reason causing two different results.

The solvent of propofol is intralipid, which is reported to have the anti-inflammatory effect in many literatures and can reduce the release of inflammatory factors [34]. Thus, after comparison of intralipid between the in vivo study and the in vitro study of this experiment, it has been found that lipid emulsion can neither inhibit the release of inflammatory factors, nor activate the expression of Annexin A1.

However, it still needs to be investigated whether Annexin A1 is the key anti-inflammatory protein of propofol and whether the anti-inflammatory effect of propofol is realized through inhibiting phosphorylation of p38. To this end, RNAi technology was applied to detect the impact of propofol on phosphorylation of p38 and pro-inflammatory factors (TNF-α, IL-1β, IL-6) under LPS stimulation after inhibition of Annexin A1 in THP-1. The results showed that propofol had no impact on phosphorylation of p38 and pro-inflammatory factors (TNF-α, IL-1β, IL-6) under LPS stimulation following inhibition of Annexin A1, which is consistent with the result that Annexin A1 can negatively regulate IL-6 through inhibiting p38MAPK, as recently reported by Yang YH [37]. The above results fully confirm that the anti-inflammatory effect of propofol is realized through inhibiting phosphorylation of p38 after up-regulation of the expression of Annexin A1 in mononuclear cells. It can be said that Annexin A1 is a key signaling molecule in the anti-inflammatory mechanism of propofol.

It is reported that Annexin A1 is mainly regulated by glucocorticoids, which plays an important role in the signal transduction pathway of glucocorticoid-mediated anti-inflammatory response and inhibits neutrophil mobilization in several experimental animal models [27]. That is to say, glucocorticoids can also activate Annexin A1. In animal experiments, it has been found that the expression of Annexin A1 was up-regulated in the propofol group, which might be due to propofol or glucocorticoids in rats' bodies. Activation of Annexin A1 by propofol is also detected in THP-1 in extracellular cells, indicating that Annexin A1 is activated by propofol, which is irrelevant with glucocorticoids.

Our study design did not indicate how propofol regulates the Annexin A1, although we demonstrated the effect of propofol on regulating Annexin A1 during LPS inflammation in vivo and vitro, which is our next research content. In addition, propofol also has anti-oxidative effect, what is this effect on Annexin A1 expression is unknown. For our experimental model in the paper, we mainly investigate effects of propofol on Annexin A1 expression during pathophysiological process, on p38 phosphorylation through Annexin A1 and on the release of inflammatory factors in endotoxemia caused by LPS. While it a very interesting and meaningful idea that antioxidant effect of propofol in this process acts to what extent. As inflammation and oxidative stress are essentially inseparable, oxidative stress can cause inflammation, and large amounts of oxygen free radicals produced in inflammatory process will also affect the inflammatory process. The relationship of the two is so close that it is difficult to completely separate the two, futher study in needed.

To conclude, the results of our study clearly show that propofol can up-regulate the expression of Annexin A1 in rats with endotoxemia and LPS-stimulated THP-1 and the increased expression of Annexin A1 can also reduce the release of inflammatory factors. Annexin A1 can also negatively regulate phosphorylation of p38. Therefore, it can be concluded that the anti-inflammatory molecular mechanism of propofol might be realized through inhibiting phosphorylation of p38 and the release of inflammatory factors following up-regulation of Annexin A1.

## Methods

### Model building

Experiments were approved by the local council of ethics and performed in accordance with the Guidelines for the Care and Use of Nanfang Hospital. To in accordance with the guidelines of the International Association for the Study of Pain as published in Pain 1983; 16:109–110, all the operation were done, after animals were anesthetized with urethane, so animals did not feel pain or discomfort during the experiments and the minimum possible pain or stress had been imposed on the animals. Animals adopted from the experimental animal center of Southern Medical University. Eighteen Male Sprague-Dawley rats weighing between 180 and 210 g anesthetized with urethane (1.0 g/kg) i.p. were divided into control, LPS (from Escherichia coli, sigma, USA), propofol(commercial name Diprivan, Astrazeneca, UK), LPS+propofol groups, intralipid group(sino-swed pharmaceuticalcorp.ltd, China) and LPS+intralipid group. In the LPS group, rats were infused with Intralipid® immediately following 10 mg.kg-1 LPS i.v., and in the LPS+propofol group rats were injected with LPS followed by propofol at 10 mg.kg-1h-1. In the intralipid group, rats received a continuous infusion of 10% Intralipid®(10 mg.kg-1h-1) via the femoral vein to control for EDTA present in the lipid diluent of propofol, and in the LPS+intralipid group rates were injected with LPS followed by intralipid at 10 mg.kg-1h-1. In the control group, rats were injected the same amount balanced saline. After 6 h, 3–4 ml of blood from the carotid artery of each rat was collected into Eppendorf tubes and the rats were sacrificed thereafter.

### Isolation of leukocytes from rat peripheral blood

3.0 ml HISTOPAQUE 1083(Sigma) were Added in a 15 ml centrifuge tubes, then 3.0 ml whole blood were carefully layered onto the HISTOPAQUE 1083 surface, Centrifuging at 400×g for exactly 30 minutes at room temperature. After centrifugation, the upper layer to within 2–3 mm of the opaque interface contained the mononuclear cells. Carefully aspirated the upper layer with a Pasteur and discarded it. Carefully transferd the opaque interface, containing the mononuclear cell band, with a Pasteur pipet, into a clean 15 ml conical centrifuge tube. Then 10 ml isotonic PBS were added to to the monuclear cells Mixing the tube by gentle inversion several times and Centrifuge at 250×g for 10 minutes. Aspirated the supernatant and discarded it. After resuspending the cell pellet with 0.5 ml of isotonic PBS, additional 4.5 ml of isotonic PBS were added to Centrifuge at 250×g for 10 minutes. Aspirated the supernatant and discarded it. Repeated this step 2–3 times to remove any remaining HISTOPAQUE 1083 from the mononuclear cells. After the final wash, the cells were 300 µL of lysis buffer consisting of 7 Murea, 2Mthiourea, 4% CHAPS, 65mMDTT and 2% Pharmalyte(pH3-10, GE HealthCare, Piscataway, NJ) by sonication on ice. The lysates were cleared by centrifugation at 12 000 rcf for 30 min at 4°C twice. Subsequently, the protein concentration of the supernatants was determined by the modified Bradford method and aliquots of the protein samples were stored at −80°C.

### 2-D electrophoresis

Immobiline Dry strip (pH 4–7, length 24 cm, GE Healthcare) was rehydrated with 1500 µg protein in 450 ml rehydration buffer containing 7 M urea, 2 M thiourea, 4% CHAPS, 65 mM DTT, 20 mM Trizma base, 1% IPG buffer and 0.002% bromophenol blue for 14 h at room temperature. Isoelectric focusing (IEF) was performed using the Ettan IPGphor 3 IEF System (GE Healthcare, USA) for a total of 70 kVh. The strip was then subjected to two-step equilibration in a buffer containing 6 M urea, 20% glycerol, 2% SDS and 50 mM Tris–HCl (pH 8.8) with 2% w/v DTT for the first step, and 2.5% w/v iodoacetamide for the second step. The second-dimension SDS-PAGE (12% T, 260×200×1.5 mm3) was carried out using a Ettan DALTsix Large Vertical system (Amersham, USA) according to the following procedures: 45 min at a constant power of 5 watt followed by 20 watt per gel until the bromophenol blue front reached the bottom of the gel. Subsequently the gels were stained with 0.12% w/v Coomassie Brilliant Blue G250. Each group was run in triplicate to minimize run-to-run variation. The coomassie blue-stained protein 2D gels were scanned using an Amersham Biosciences Imagescanner and analyzed using DeCyder software package (GE Healthcare, USA).

### In-gel digestion

Protein spots were excised from gel with an operating knife blade, destained twice with 30 mM potassium ferricyanide and 100 mM sodium thiosulfate (1∶1 v/v) and then equilibrated in 100 mM NH_4_HCO_3_ to pH 8.0. After dehydrating with ACN and drying in N2 at 37°C for 20 min, the gel pieces were rehydrated in 10 µl trypsin solution (12.5 ng/µl in 50 mM NH_4_HCO_3_) at 4°C for 30 min and incubated at 37°C overnight. Peptides were extracted twice using 0.1% TFA in 60% CAN and dried with the RCT60 (Jouan, France).

### MALDI-TOF-MS identification

The peptide mixtures were solubilized with 0.1% TFA and desalted with C18 ZipTip (Millipore, USA). The peptide was then eluted by saturated a-cyano-4-hydroxy-trans-cinnamic (CHCA) solution in 0.1% TFA/60% acetonitrile as the matrix and analyzed using 4800 MALDI TOF/TOF Analyzer (Applied Biosystems, USA). Mass spectra were internally calibrated with angiotensin I (Mr: 1296.6853).

### Protein identification and database searching

Protein identification using peptide mass fingerprinting (PMF) and peptide sequence tag (PST) was performed by the MASCOT search engine (http://www.matrixscience.com/, MatrixSicence Ltd., London, UK) against the SwissProt protein database. The errors in peptide masses were in the range of 50 ppm. One missed tryptic cleavage site per peptide was allowed during the search. Proteins matching more than four peptides and with a MASCOT score higher than 64 were considered significant (P<0.05). Carboamidomethylation of cysteine was selected as the fixed modification and oxidation of methionine as the variable modification. Protein identification results were filtered with GPS software.

### Cell culture and reagents

The human monocytic THP-1 cells from the American Type Culture Collection(ATCC) were cultured in RPMI1640(Gibco BRL, Grand Island, NY, USA) supplemented with 10% fetal calf serum (FCS)(Gibco BRL, Grand Island, NY, USA) in 75-cm^2^ flasks at 37°C in a humidified atmosphere of 5% CO_2_. The cultured THP-1 cells were randomly assigned to one of the following six groups: Group 1: cells in untreated group(control) was cultured for 6 h in the absence of propofol and LPS; Group 2: treated with 10 µg/ml LPS for 6 h; Group 3: treated with 20 µg/ml propofol for 6 h; Group 4: treated with 10 µg/ml LPS and 20 µg/ml propofol for 6 h; Group 5: treated with 20 µg/ml intralipid(the solvent for propofol) for 6 h; Group 6: treated with 10 µg/ml LPS and 20 µg/ml intralipid for 6 h;

### Transient transfection of Annexin A1 shRNA into THP-1 cells

shRNA of Anneinx A1 were synthesis by Gene Pharma RNAi Cmpany. The sequence were: 5′ CCCUGGAUGAAACACUUAATT 3′; 5′GCAGGAAUAUGUUCAAACUTT 3′; 5′GCCUUGCAUAAGGCCAUAATT 3′. The shRNA and negative control shRNA were transfected into THP-1 cells by electroporation. THP-1 cells (1×10^3^) were washed in PBS and then centrifuged and resupended in 100 ul R buffer; 100 nM shRNA was then added. Electroporation was performed using a NEON(1500 V, 20 ms, 2 N). After discharging, cells were transferred into 500 ul containing 10% fetal serum medium and further incubated 24 h under atmospheric conditions of 95% air-5% CO_2_ at 37°C.

### Western Blot Analysis

Annexin A1 and phospho-p38MAPK(Thr180/Tyr182) in the mononuclear cells of rats and THP-1 cells were detected by western blot analysis. The mononuclear cells of rats and THP-1 cells were lysed on ice in 300 µl cell lysis buffer [1× PBS, 1% NP40, 0.1% sodium dodecyl sulfate (SDS), 5 mM EDTA, 0.5% sodium deoxycholate, and 1 mM sodium orthovanadate] with protease inhibitors. Protein concentration was determined by the modified Bradford method. Equal amounts of proteins were separated electrophoretically on 12% SDS/polyacrylamide gels and transferred onto polyvinylidene difluoride membranes (PVDF) (Amersham Pharmacia Biotech, Piscataway, NJ). For Annexin A1 detecting, the membrane was probed with anti-Annexin A1 rabbit polyclonal antibody (1∶2000; Cell Signaling Technology) and horseradish peroxidase-conjugated anti-rabbit immunoglobulin G (1∶2000; Cell Signaling Technology, Danvers MA); For Phospho-p38MAPK detecting, the membrane was probed with anti-Phospho-p38MAPK (Thr180/Tyr182) rabbit polyclonal antibody (1∶1000; Cell Signaling Technology, Danvers MA) and horseradish peroxidase-conjugated anti-rabbit immunoglobulin G (1∶2000; Cell Signaling Technology, Danvers MA); For total p38MAPK detecting, the membrane was probed with anti-p38MAPK rabbit polyclonal antibody (1∶1000; Cell Signaling Technology, Danvers MA) and horseradish peroxidase-conjugated anti-rabbit immunoglobulin G (1∶2000; Cell Signaling Technology, Danvers MA); For β-actin detecting, the membrane was probed with anti-β-actin (HRP-Conjugate) rabbit monoclonal antibody (1∶1000; Cell Signaling Technology, Danvers MA).

### ELISA

To test the amount of IL-1β, TNF-α and IL-6 in the sera of another 24 SD rats and the supernatant of THP-1 cells were randomized into same treatment groups. Measurements were carried out using commercially available ELISA kits (Rat or Human IL-1β ELISA Kit eBioscience, Rat or Human TNF-α ELISA Kit Uscnlife, Rat or Human IL-6 ELISA Kit Thermo Scientific), in accordance with the manufacturer's instructions. All sera and supernatant were stored at −80°C before they were measured. Both standards and samples were run in duplicate.

### Reverse transcriptase-polymerase chain reaction

Total RNA was extracted from treated THP-1 cells using TRIzol reagent (Gibco BRL, Grand Island, NY) and subsequently underwent reverse transcription, according to the manufacturer's protocol. IL-1β, TNF-α, IL-6 and β-actin were amplified using polymerase chain reaction (PCR) with gene-sepcific primers (Table 2). PCR products were visualized on 2% agarose gels containing ethidium bromide. β-actin was the internal control. The relative quantity of PCR products is expressed as fold increase relative to PBS controls.

**Table 2:** Primers pairs used in this study.

Factor	Primer sequence (5′-3′)
IL-1β	F-CCCAAGCAATACCCAAAGAA
	R-CATCAGAGGCAAGGAGGAAA
IL-6	F-GACAGCCACTCACCTCTTCA
	R-CATCTTGGAAGGTTCAGGTTGT
TNF-α	F-TGACAAGCCTGTAGCCCATG
	R-CAAAGTAGACCTGCCCAGAC
β-actin	F-TGTCCCTGTATGCCTCTGGT
	R-GATGTCACGCACGATTCC

### Statistical Analysis

All the data was tested for normal distribution before statistical analysis and the statistic analysis were carried out using the SPSS13.0. All data was and expressed as mean ± S.D. Multiple groups were compared using analysis of variance (ANOVA) followed by Student-Newman-Keuls post hoc procedure. Changes were identified as significant when P<0.05.
